# Development of an enzyme immunoassay to measure urinary and faecal 5α-androst-16-en-3-one in pigs

**DOI:** 10.1016/j.mex.2023.102178

**Published:** 2023-04-13

**Authors:** Vinod Kumar, Govindhaswamy Umapathy

**Affiliations:** Laboratory for the Conservation of Endangered Species (LaCONES), CSIR-Centre for Cellular and Molecular Biology (CCMB), Hyderabad, Telangana 500048, India

**Keywords:** Androstenone, Boar taint, Pheromone, Enzyme immunoassay, Antibody production, Faeces, Urine, Development of an enzyme immunoassay for detection of faecal and urinary 5α-androst-16-en-3-one in pigs

## Abstract

Androstenone, a volatile steroid that possesses pheromonal activity, is responsible for boar taint, sexual interactions, and reproduction in pigs. A wide range of analytical methods has been developed to quantify and detect androstenone in adipose tissue and blood, which are invasive procedures. Therefore, the present study aimed to develop a non-invasive method to detect and quantify the androstenone. We produced group-specific polyclonal androstenone antibody to standardize and validate an enzyme immunoassay to measure faecal and urinary androstenone in Yorkshire boars and sows. Parallelism was performed to determine the immunoreactivity between faecal and urinary immunoreactive androstenone and respective antibody. In boars, urinary and faecal androstenone concentrations were higher on the day of mounting and copulation with sows. In sows, we also measured faecal progesterone metabolites to confirm the oestrus and mating. Faecal androstenone concentrations were peaked on the day of oestrus and mating in sows. Our results suggest that androstenone could be detected and quantified in faecal and urine samples of boars and sows.

• Developed an enzyme immunoassay for measuring 5α-androst-16-en-3-one as a marker of boar taint and sex pheromone in urine and faeces of pigs
• Detection of 5α-androst-16-en-3-one using a non-invasive method

## Graphical abstract

**Figure:**
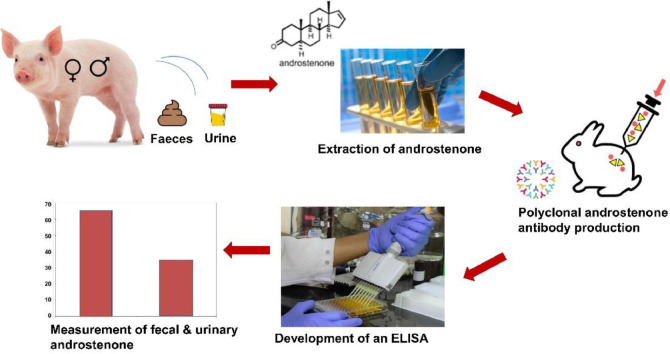

Specifications Table

**Table:** 

Subject area:	Agricultural and Biological Sciences

More specific subject area:	Boar taint and reproduction in pigs
Name of your method:	Development of an enzyme immunoassay for detection of faecal and urinary 5α-androst-16-en-3-one in pigs
Name and reference of original method:	Claus, R., Lacorn, M., & Ostertag, C. (2008). An improved microtitre enzyme immunoassay to measure the boar taint steroid 5α-androst-16-en-3-one in blood plasma of pigs. Meat science, 80(3), 934–938.
Resource availability:	Yes, for hormones (https://www.steraloids.com), Freund's complete/incomplete adjuvants https://www.sigmaaldrich.com

## Introduction

Androstenone (5α-androst-16-en-3-one) and androstenol (5α-androst-16-en-3α-ol) are naturally occurring 16-androstenes steroids first described by Prelog and Ruzicka [1] in 1944 from testicular tissue, as produced by Leydig cells of testes in mature boar. Subsequently, both steroids were regarded as pheromones contributed to urine like smell (androstenone) and musky odour smells (androstenol) in boar adipose tissue [2,3], which play a crucial role in altering the reproductive behaviour in gilts/sows [4]. However, androstenone is responsible for boar taint accompanied by high levels of skatole (3-methylindole) and lesser amount of indole (2,3-benopyrol) [[5], [6], [7]]. Boar taint is an unpleasant odour in pork, which makes their meat products undesirable to consumers [8]. Androstenone is stored in adipose tissue in much higher concentrations than other testicular steroids. Besides, it is released in the boar saliva through salivary gland and acts as pheromone [9]. Adult boars are frequently castrated before they reach sexual maturity to control the levels of androstenone [10]. Further, castration leads to animal cruelty and raising major concerns about animal welfare. However, a study suggested that the androstenone and skatole levels could be controlled by nutritional and genetic selections in the diet and breeding individuals, respectively [11]. Presently, assays are available to measure androstenone in adipose tissue and blood plasma [11,12].

These methods require collection of blood plasma, fat tissue, and saliva, which need restraining or handling of animals. Further, repeated blood or saliva sampling causes stress to the animals. As an alternative method, non-invasive androstenone estimation using faeces and urine is a more practical and feasible method that requires minimal or no contact with the animal [13]. Thus, developing an enzyme immunoassay (EIA) for faecal and urinary androstenone would be pertinent for animals and farmers. Faeces and urine are the matrix of metabolized 16-androstenes group steroids in relation to free or sulphoconjugated steroids present in the blood. Steroids are metabolizing in the liver, kidney and testes through phase I reactions. These compounds then further conjugated by phase II enzymes including sulfotransferases (SULTs) or uridine diphosphate glucuronosyltransferases (UGTs), resulting metabolites are turned out to be more water soluble and excrete through urine and via bile into the faeces [14], [15], [16], [17]. Therefore, faeces and urine provide cumulative excretion of steroid metabolites over several hours than blood plasma.

In the past, androstenone was measured in the fat, plasma, and saliva using multidimensional gas chromatography/mass spectrometry (MDGC/MS) [18], High-performance liquid chromatography (HPLC) with fluorescence method [11], liquid chromatography-multiple mass spectrometry [19], Ultra-High-Performance Liquid Chromatography coupled with high-resolution mass spectrometry [20], radioimmunoassay (RIA) [21], enzyme immunoassay (EIA) [22,11], and fluoroimmunoassay (FIA) [23], and Colorimetric assay [24]. The present study aimed to develop and standardize an enzyme immunoassay (EIA) for measuring androstenone in faeces and urine of pigs.

## Material and methods

### Sample collection

A total of 30 faecal and 20 urine samples were collected from 05 Yorkshire pigs (02 males and 03 females) from livestock farm complex, College of Veterinary Science, Rajendranagar, Hyderabad. The facility maintains all data on reproductive fitness, pregnancy and oestrous cycle of all animals. We collected samples during oestrus (when mountings and copulations observed) as androstenone levels are known to be higher in matured boars [24]. To prevent urine contamination and human handling, extreme caution was employed while collecting faecal samples. Faecal samples were collected following urine sample (opportunistically) collection in the morning hours, but before 10.00 am. The samples were collected in an air tight tube and stored in −20 °C until further analysis. Overall, the mean age of animals was 2.94 ± 0.198, and the age ranged between 2.4 and 3.5 years.

### Sample extraction

Faecal samples were extracted according to a previously described procedure [25]. Approximately 0.5 g of wet faeces was weighed, and 3 ml of 80% methanol was added. The samples were stored overnight at 4 °C after being vortexed for 30 min at room temperature. The sample was centrifuged the next day at *3300 g* for 20 min, transferred to a new tube, and stored at −20 °C until further analysis.

### Antibody production

Polyclonal antibody was produced against immunoreactive androstenone to develop the sensitive indirect competitive enzyme immunoassay. For this purpose, two adult New Zealand white rabbits were immunized with BSA-conjugated 16, (5α)-androsten-3-one-carboxymethyloxime (Steraloids, USA). The first immunization dose was prepared using Freund's complete adjuvant (Sigma-Aldrich Chemical Company, St-Louis, MO) with an equal volume of 1 mg/ml of 16, (5α)-androsten-3-one-carboxymethyloxime: BSA conjugate dissolved in 0.9% saline and emulsified until it becomes thick white solution. Four to six sites on the back of the rabbit received subcutaneous injections of the emulsified fluid. After two weeks of first injection, booster shots (0.5 mg/ml) were administered with Freund's incomplete adjuvant following every two weeks on day 28, 42, 56, and 70. Blood was collected every week of the booster dose on day 35, 49, 63 and 76 [26,27]. Serum was isolated from blood by incubating at room temperature for 4–6 hrs. (or overnight at 4 °C) and vortexed at 4000 rpm for 10 mins. Protein-A affinity chromatography (Pierce, India) was used to purify the polyclonal Immunoglobin (IgG) antibodies. The final concentration of anti-androstenone antibody was estimated to be 15 mg/ml and stored at −80 °C after the aliquots of purified antibody were pooled. The optimal antibody dilution for the androstenone assay was determined using the checkerboard titrations.

### Androstenone EIA and procedure

Faecal and urinary androstenone were quantified using polyclonal anti-androstenone antibody which was diluted to 1:25,600, androstenone standards (1000–3.9 ng/ml), BSA-conjugated 16, (5α)-androsten-3-one-carboxymethyloxime (1 mg/ml) and horseradish peroxidase (HRP)–conjugated goat anti-rabbit IgG secondary antibody (GeNei) diluted to 1:10,000.

The indirect competitive ELISA procedure for 5α-androst-16-en-3-one was carried out as previously reported [27], [28], [29]. The 96 well microtiter plate (Nunc Maxisorp; Immuno plate, Denmark) was coated with 100 µL of 1 µg conjugate/mL of 16, (5α)-androsten-3-one-carboxymethyloxime: BSA (diluted in coating buffer, 0.05 M sodium bicarbonate buffer, pH 9.6), placed in moist chamber and covered with cling wrap and incubated overnight at 4 °C. The plate's contents were removed, and an automated ELISA washer (Elx50, BioTek, USA) was used to wash the plate four times with wash buffer (0.15 M NaCl, 0.05% Tween 20). After blocking the plate with 200 µl of blocking buffer, it was incubated at 37 °C for one hour. The plate's contents were discarded, then it was blotted and allowed to air dry. Subsequently, 50 µl of diluted faecal sample (final dilution 1:4 in EIA buffer) and 50 µl of 5α-androst-16-en-3-one standards followed by 50 µl of diluted 5α-androst-16-en-3-one antibody was added and incubated at 37 °C for 90 min. The plate was again washed with wash buffer, blotted, and 100 µl of horseradish peroxidase (HRP) conjugated goat anti-rabbit IgG secondary antibody was added in each well and incubated at 37 °C for 90 min. The plate was washed as mentioned above, and 100 µl of substrate solution TMB/H_2_O_2_ (Tetramethylbenzidine/Hydrogen peroxide, Genei) was added and incubated in dark for 5–10 mins (for colour development). The reaction was stopped with 50 µl of stopping solution (1 N HCL), and the ELISA reader (Thermo Multiskan Spectrum Plate Reader, version 2.4.2, Thermo Scientific, Finland) read the absorbence at 450 nm.

### Cross-reactivity of androstenone antibody

The 5α-androst-16-en-3-one antibody cross-reactivity with C19 structurally close related and other steroids was estimated using half displacement method as described by Abraham [30,27]. The androstenone antibody cross-reacted with androstenone (100%), 4, 16-androstadien-3-one (164%), androst-4-ene-3,17‑dione (32.7%), 5α-androst-16-en-3α-ol (12%), Progesterone (9.44%), 5,16-Androstadien-3β-ol (6.47%), 5α-dihydrotestosterone (2%), testosterone (3.33%), 5-androstenediol (<1%), and oestradiol (<1%) [29].

### Progesterone EIA

Faecal progesterone metabolites were quantified using previously developed EIA [31], [32], [33], [34]. In progesterone EIA, monoclonal antibody (CL425, provided by Dr. Coralie Munro, University of California, USA) was diluted to 1:6000, HRP (Horseradish peroxidase) conjugated progesterone diluted to 1:100,000 and progesterone standards (200 pg/well to 0.39 pg/well). The progesterone antibody showed 100% cross-reactivity with progesterone and other 5α and β-reduced pregnane [32]. The procedure of progesterone EIA was performed as described previously [31], [32], [33], [34], [35].

### Creatinine assay for urinary androstenone

Creatinine is a byproduct of protein metabolism and excreted in urine at a constant rate. Therefore, creatinine is used as urinary hormone concentration indexed to the concentration of creatinine to control the variation of water volume in urine samples [36]. It was measured by using modified method of Jaffe reaction previously described by Taussky [37]. Creatinine concentration show amount of time over which hormones are metabolizing and excreting in the urine [38]. The procedure for creatinine assay was performed as described earlier [33]. Urinary hormone concentrations are expressed as nanograms per micrograms of creatinine.

### Data analysis

Urinary androstenone concentrations were calculated from the standard curve and expressed in ng/µg of creatinine for each sample. The data of faecal androstenone and progesterone was presented in mean ± SEM and ng/g of wet faeces. The Mann–Whitney U test (M–W test) was used for testing differences in faecal androstenone concentrations during mounting and copulation than post copulation in males.

## Results & discussion

### Validation of androstenone EIA

Androstenone EIA was validated by using parallel displacement curves to demonstrate parallelism between the pooled serial dilution of faecal extracts of pigs (endogenous) and respective standards (exogenous). Parallelism was performed to determine the optimum dilution of faecal and urinary androstenone at 50% binding and immunoreactivity of endogenous androstenone with the corresponding antibody used in the assay. The most sensitive range of calibration curve of androstenone standard was found between 1000 ng/ml and 3.9 ng/ml. The lowest concentrations of androstenone would require an addition of faecal extracts without diluting in EIA buffer. The sensitivity of the androstenone antibody was calculated at 90% binding and estimated to be 3.9 ng/ml. Recovery and accuracy of the assay were determined by spiking the known concentrations of unlabelled exogenous androstenone in faecal extracts and urine sample. Recovery validation was performed to test the potential interference substance in the urine and faecal extracts. Recovery of known amount of unlabelled androstenone was 67.03 ± 2.6%, 79.63 ± 8.1% and 74.26 ± 2.6% for female faeces, male faeces, and urine samples, respectively (Table 1). The correlation (*r^2^*) and slope (*m*) value for the exogenous androstenone was *r^2^* =0.99, *m* = 0.63; *r^2^* = 0.96, *m* = 0.59; and *r^2^* = 0.99, *m* = 0.68 for female faeces, male faeces and male urine samples. The intra and inter-assay coefficient of variation (CV) for measurement of biological samples were 8.56% and 14.72% (*n* = 5 plates).

**Table 1:** Recovery of androstenone from exogenous spiked in faeces and urine samples of pigs.

S. No	Biological samples	Correlation (*r^2^*)	*S*lope (*m*)	Recovery (%)
1	Male faeces	0.96	0.592	79.63 ± 8.1
2	Female faeces	0.99	0.638	67.03 ± 2.3
3	Male urine	0.99	0.682	74.26 ± 2.6

### Measurement of faecal and urinary androstenone in boars

**Fig. 1:**
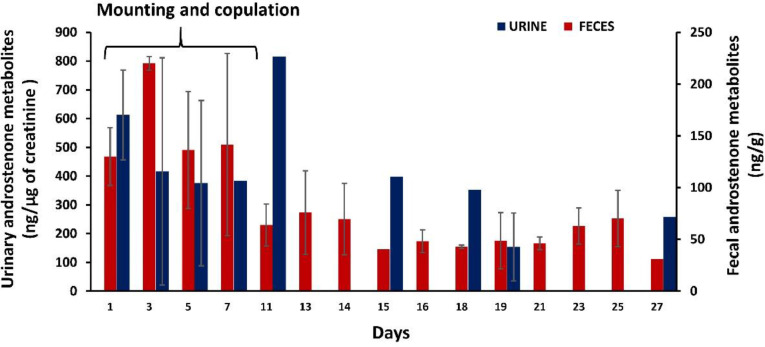
Profile of urinary and faecal androstenone in boars during mounting/copulation and mating with sows (*n* = 2).

In boars, the mean urinary androstenone concentrations ranged from 153.66 ± 117.78 ng/µg of creatinine to 815.69 ng/µg of creatinine. The higher urinary androstenone concentrations were observed during mounting and copulation with sows than in the post copulation period. The androstenone concentrations were not significantly different between copulation/mounting and post copulation days (Mann-Whitney U test: *W* = 21, *p* = 0.41; Fig. 1). The mean faecal androstenone concentrations ranged from 30.85 ng/g to 220.01 ± 6.59 ng/g (Fig. 1), and higher androstenone concentrations were observed during mounting and copulation than in the post copulation days (Mann-Whitney U test: *W* = 66, *p* = 0.001; Fig. 1).

### Measurement of faecal androstenone and progesterone metabolites in sows

**Fig. 2:**
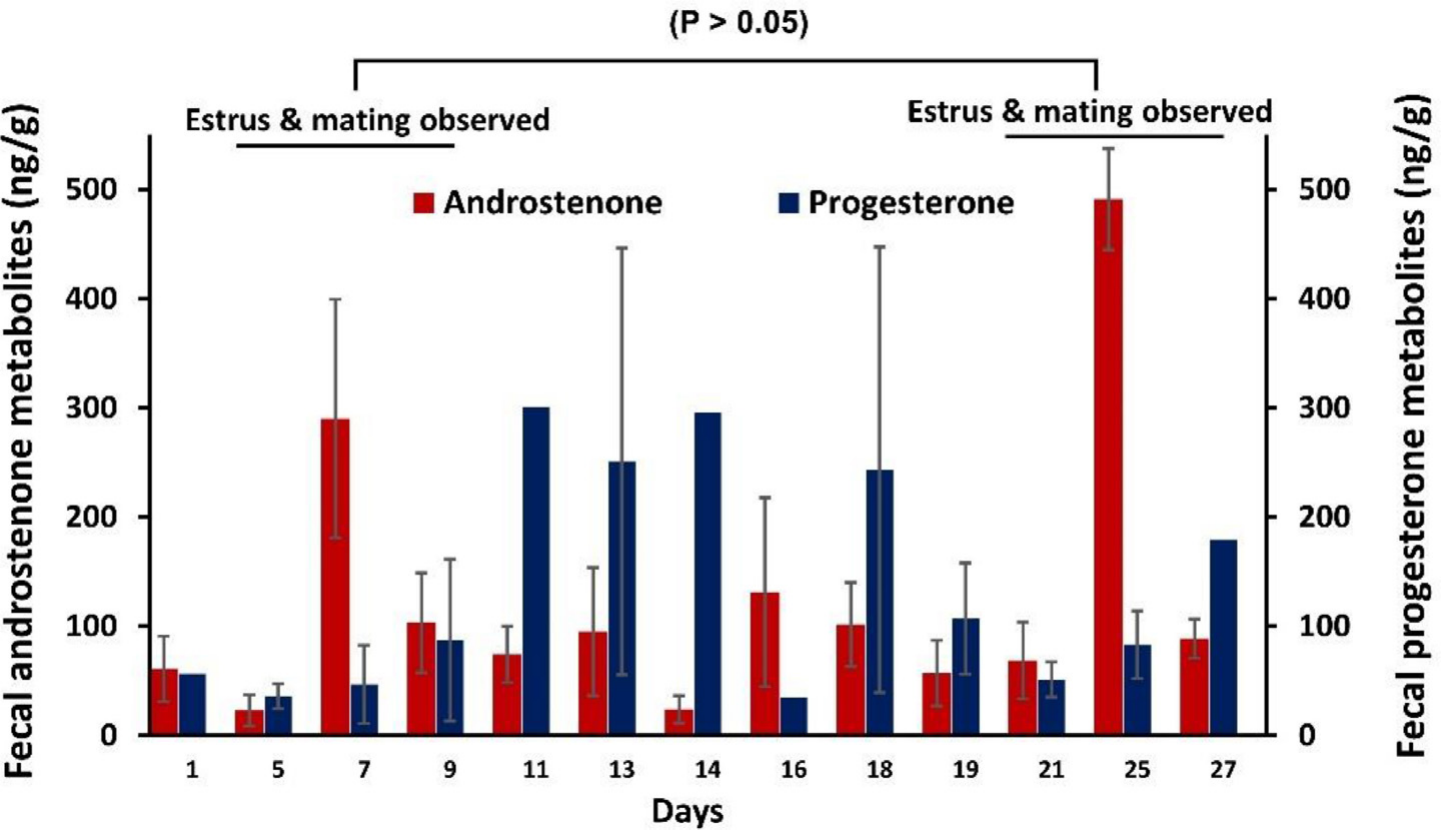
Profile of faecal androstenone and progesterone metabolite during oestrus and mating in sows (*n* = 3).

All females were observed with oestrus and mating behaviour during faecal sample collection. In sows, the mean faecal androstenone concentrations ranged from 22.94 ± 14.21 ng/g to 491.18 ± 46.37 ng/g during the study period (Fig. 2). The mean faecal androstenone concentrations were significantly higher during mating period than in the post mating period (Mann-Whitney U test: *W* = 13, *p* = 0.001; Fig. 2). However, mean androstenone concentrations were not significantly different between first and second oestrous (Mann-Whitney U test: *W* = 24, *p* = 0.470; Fig. 2). Overall, the mean progesterone metabolites concentrations ranged from 34.91 ng/g to 300.87 ng/g. Faecal progesterone metabolites concentrations found to be lower during mating and oestrus in all sows (*n* = 3) (Fig. 2).

The present study demonstrates the development and standardization of enzyme immunoassay for the detection of androstenone in boars and sows using non-invasive samples. The polyclonal antibody produced against androstenone is able to measure conjugated and unconjugated androstenone metabolites (16-androstenes) present in faeces and urine of pigs. The androstenone antibody had good cross-reactivity with other C-19 closely related androstenone precursor (4, 16-androstadien-3-one) and metabolites (Androstenol) in faecal and urine samples.

Previously, developed enzyme immunoassays were standardized for the detection of androstenone in adipose tissue [11] and blood plasma [22]. In this study, we developed an EIA for the detection of androstenone in urine and faecal samples of pigs using a non-invasive method. We confirmed the presence of faecal and urinary androstenone and immunoreactivity using parallel displacement curves between faecal and urinary androstenone, and their respective standards. We also validated androstenone EIA by performing a recovery or accuracy check using known concentrations of exogenous androstenone to determine any potential interference hindrance substance in the faecal and urine samples. The developed androstenone EIA has higher antibody sensitivity, low inter and intra-assay coefficient of variation, and high recovery rates.

Bonneau and Terqui [39] have shown that 51% radiolabeled ^3^H androstenone was excreted through urine within a week along with a small amount in faeces. In this study, we have shown that androstenone excretion in faeces and urine, though we could not assess the nature of androstenone metabolites whether is conjugated or unconjugated form in the faeces and urine. A further study is required to find more details. Previous reports suggest androstenone is probably catabolized in liver [40], and eliminated in uterine mainly as an-β glucuronides and an-α conjugates and leads to more unidentified polar steroids found in plasma and urine of mature boar [41], [42], [43]. However, other metabolites other than an-α and an-β have been reported previously in boar plasma [43], and human urine [44]. Conjugated steroids have high molecular polarity that improves their solubility in aqueous solution [45]. Therefore, 80% of methanol and 20% of water is a suitable ratio for the extraction of conjugated and unconjugated forms of androstenone in faeces.

Although, faecal and urinary androstenone concentrations ranged widely and significantly elevated during matings as compared to post matings in boars (Fig. 1). Of the two boars, one of them was found to be aggressive and dominance, which also had high levels of androstenone during mounting and copulation compared to other one. A similar result was observed by Giersing et al. [46]. In sows, faecal androstenone concentrations were peaked on the day of mating and declined during post mating days. However, previous reports have showed that no significant difference in plasma 5α androstenone during oestrus [47]. Faecal progesterone metabolites showed a clear peak during the post-mating period, which is the evidence of luteal phase in three sows, as reported previously [48].

The developed non-invasive ELISA method has more advantages over conventional methods, including slaughtering the boar to harvest the fat or adipose tissue and restraining the boar to collect the blood samples, etc. Invasive methods raised concern about animal welfare and cruelty. Therefore, the present method is most appropriate way to measure androstenone using a non-invasive method. Further, the conventional method uses strong organic solvents for the extraction of androstenone from fat tissue [49], with additional step such as solvent-solvent extraction and then evaporation, resulting in the loss of volatile androstenone. These steps are not required for non-invasive ELISA, where water-miscible solvents are used, particularly methanol [50] and ethanol [51]. The potential limitation of non-invasive ELISA is that urine and faeces may have both conjugated/unconjugated metabolites of androstenone which might interfere in the measurement of native androstenone. However, further study is required to assess the nature of urinary and faeces androstenone.

## Conclusion

Non-invasive androstenone monitoring has several advantages over invasive method as blood collection causes stress to the animal pigs. Therefore, the development of non-invasive method of ELISA would be more beneficial in measuring faecal and urinary androstenone as a marker of boar taint and sex pheromone in boars and sows

## CRediT author statement

*VK and GU: Conceptualization, Methodology, Software VK: Validity tests, Data curation, Writing- Original draft preparation. VK: Visualization, Investigation. GU: Supervision and Resources. VK, GU: Writing- Reviewing and Editing.*

## Related research article

*Claus, R., Lacorn, M., & Ostertag, C. (2008). An improved microtitre enzyme immunoassay to measure the boar taint steroid 5α-androst-16-en-3-one in blood plasma of pigs. Meat science, 80(3), 934–938 10.1016/j.meatsci.2008.04.019*

## For a published article

Claus, R., Lacorn, M., & Ostertag, C. (2008). An improved microtitre enzyme immunoassay to measure the boar taint steroid 5α-androst-16-en-3-one in blood plasma of pigs. Meat science, 80(3), 934–938 10.1016/j.meatsci.2008.04.019

## Declaration of Competing Interests

The authors declare that they have no known competing financial interests or personal relationships that could have appeared to influence the work reported in this paper.

## Data Availability

Data will be made available on request.
